# Integration of metabolome and transcriptome reveals flavonoid accumulation in the intergeneric hybrid between *Brassica rapa* and *Raphanus sativus*

**DOI:** 10.1038/s41598-019-54889-2

**Published:** 2019-12-04

**Authors:** Libin Zhang, Chuang Ma, Hongbo Chao, Yan Long, Jiangsheng Wu, Zaiyun Li, Xianhong Ge, Heng Xia, Yongtai Yin, Jacqueline Batley, Maoteng Li

**Affiliations:** 1 College of Life Science and Technology, Huazhong University of Science and Technology, Wuhan, China; 2 State Key Laboratory of Crop Stress Biology for Arid Areas, College of Life Sciences, Northwest A&F University, Yangling, China; 3 Biotechnology Research Institute, Chinese Academy of Agricultural Sciences, Beijing, China; 4 National Key Laboratory of Crop Genetic Improvement, Huazhong Agricultural University, Wuhan, China; 5 School of Plant Biology, The University of Western Australia, Crawley, Australia

**Keywords:** Plant breeding, Plant molecular biology

## Abstract

*Brassica rapa* and *Raphanus sativus* are two important edible vegetables that contain numerous nutritional ingredients. However, the agronomic traits and nutritional components of the intergeneric hybrid of *B*. *rapa* and *R*. *sativus* remain poorly understood. In this study, we used a stably inherited intergeneric hybrid of *B*. *rapa* and *R*. *sativus* as a model to study its metabolome and transcriptome profiles. Morphological and cytological analysis showed the intergeneric hybrid had the expected chromosome number and normal meiosis behavior. Moreover, the metabolome analysis showed multiple important secondary metabolites, including flavonoids and glucosinolates, were significantly upregulated in the hybrid. Furthermore, transcriptome data revealed that the expression level of the important genes involved in phenylpropanoid and flavonoid pathways was significantly upregulated in the hybrid. Ultimately, our data indicate the intergeneric hybrid will be a valuable bioengineering resource and promise to become a new-type hybrid vegetable with great medicinal value in future.

## Introduction

Distant hybridization and allopolyploidization lead to the origin and evolution of many important crops. At present, plants of the *Brassicaceae* are one model system to study crop allopolyploidization^1,2^. *Brassicaceae* plants comprise many important species and are widely cultivated in the world. For example, *Brassicaceae* contains 372 genera and over 4000 species, including many important vegetables with great economic value^3^. *R*. *sativus* (RR, 2n = 18) is an edible root vegetable and includes many varieties with different sizes, colors, flavors and planting times. *R*. *sativus* has unique flavor due to the various endogenous chemical compounds produced by the plant^4^. *Brassica rapa* (AA, 2n = 20) is also an edible vegetable in the Brassicaceae family. Moreover, various studies show that *B*. *rapa* has very high medicinal value to moisten lungs and relieve cough and asthma^5–10^. The pharmacological activities of *B*. *rapa* and *R*. *sativus* are the result of many important chemical components, including flavonoids, anthocyanins, phenolic acids, chalcone glycosides, vitamins and so on. As a group of secondary plant metabolites, flavonoids protect plants from reactive oxygen species (ROS) that result from abiotic stress such as UV-B radiation and soil salinity^11^. In addition, flavonoids control the development of individual plant organs by acting as auxin controllers^12,13^. Although metabolic and transcriptome profiles of many vegetables in the *Brassicaceae* family including *B*. *rapa* and *R*. *sativus* have been widely studied in recent years^14–18^, metabolic and transcriptome profiles of the intergeneric hybrid of *B*. *rapa* and *R*. *sativus* are rarely reported.

The breeding of new vegetables with high nutritional quality has become a popular research topic in recent years. The metabolites are usually regarded as the end products of gene expression in plants. Some metabolomic technologies, including LC-MS and/or GC-MS, have been widely applied in the identification and assessment of the expression levels of multiple metabolites in plants^19–24^. High-throughput sequencing is widely used for the transcriptome analysis of different varieties, mutants and tissues of the Brassicaceae family. Transcriptome analysis has become an important tool to discover new signaling pathways and regulatory mechanisms of gene expression during hybridization. Recently, transcriptome sequencing has been combined with metabolomics analysis to increase the power of bioinformatics analysis^25–27^. Metabolomics analysis is a quantitative detection of all metabolites and their biochemical states in specific organisms or tissues, which provides a powerful way to study the metabolic phenotype of organisms in the study of functional genomics. Therefore, the combination of metabolomics with transcriptomic analysis has been used to explore the important genes participating in the regulatory pathways of specific metabolites in plants.

In this study, we used the intergeneric hybrid of *B*. *rapa* and *R*. *sativus* as a model to study its metabolic and transcriptome profiles. Morphological and cytological analysis first showed that the intergeneric hybrid displayed the expected chromosome number and normal meiosis behavior. We then investigated the overall metabolite landscape of *B*. *rapa*, *R*. *sativus* and their hybrid by metabolomics analysis. GC-MS and LC-MS identified a total of 623 metabolites of which 135 differentially expressed metabolites were found among *B*. *rapa*, *R*. *sativus* and the hybrid. Further analysis indicated that various phenylpropanoids, flavonoids and anthocyanins were upregulated in the hybrid compared with those in its parents. Moreover, transcriptome analysis found that the expression level of the genes that encode important enzymes involved in the phenylpropanoid and flavonoid/anthocyanin pathways was significantly upregulated in the hybrid in comparison with that in its parents. Taken together, the results demonstrated that the intergeneric hybrid of *B*. *rapa* and *R*. *sativus* was stably inherited and had more health-promoting metabolites than those of its parents, which indicate that the intergeneric hybrid has potential to become a new hybrid vegetable in the future.

## Results and Discussion

### Morphological and cytological analyses of the intergeneric hybrid of *B*. *rapa* and *R*. *sativus*

**Figure 1:**
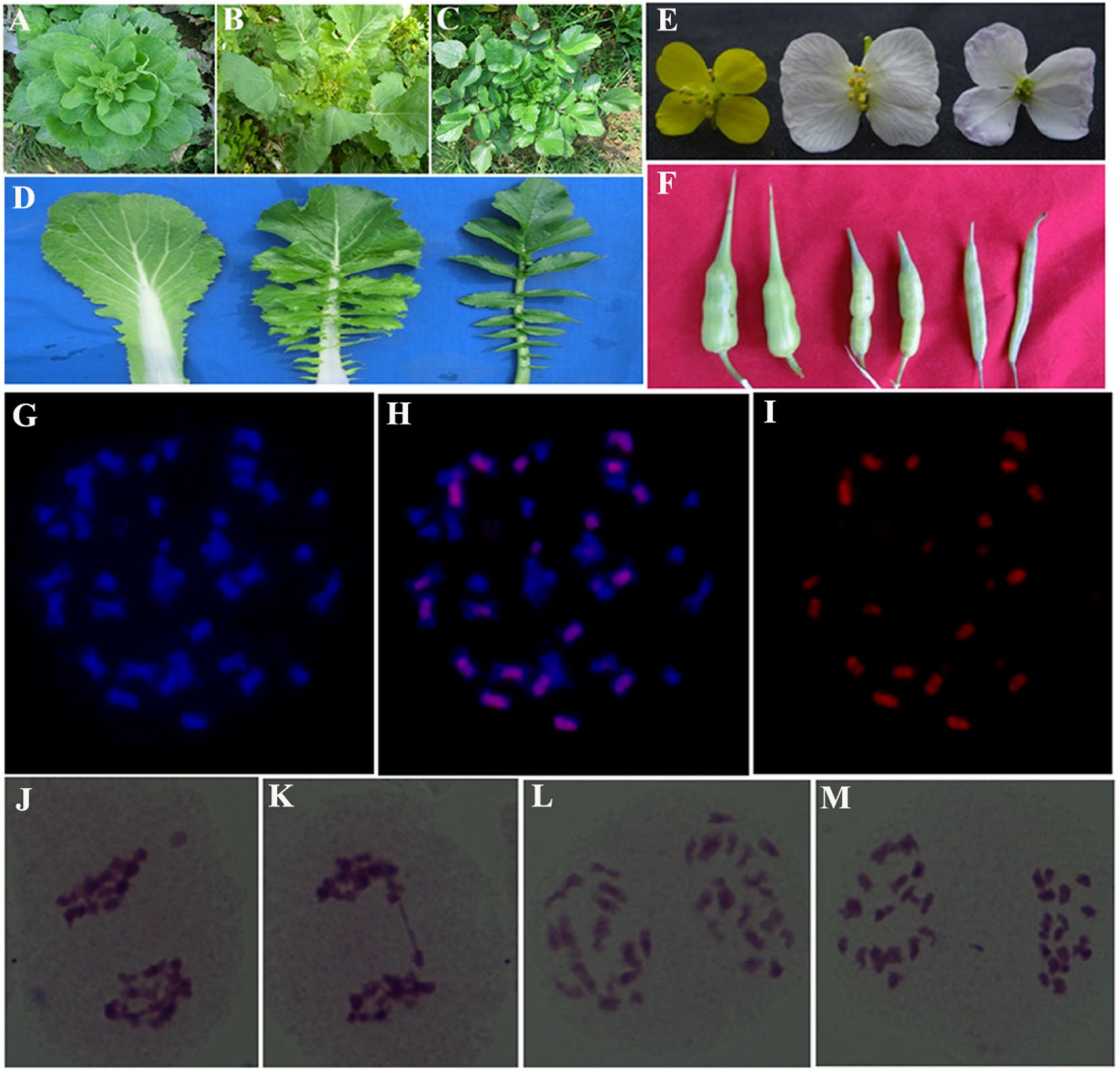
Morphological and cytological analysis of hybrid and its parents. (**A**–**F**). The morphological comparison of leaf (**D**, in maturity stage), flower (**E**) and silique (**F**) comparison of *B*. *rapa* (Left), hybrid (Middle) and *R*. *sativus* (Right), respectively. (**G**–**I**). GISH analysis of hybrid chromosomes. 18 chromosomes were successfully hybridized with DNA of *R*. *sativus* (red signal). (**J**–**M**). The meiosis analysis of hybrid ((**G,H,J**) were the cells with abnormal meiosis behavior, I was the cell with normal meiosis behavior).

The morphological analysis showed that the intergeneric hybrid of *B*. *rapa* and *R*. *sativus* exhibited intermediate characteristics between *B*. *rapa* and *R*. *sativus* with some exceptions including the color of leaves, the flower and the silique (Fig. 1A–F). Chromosome identification of the intergeneric hybrids was performed, and the plants with 38 chromosomes were selected for further studies (Fig. 1G–I). Moreover, the chromosomes in somatic cells of the hybrid were hybridized *in situ* with the genomic DNA of *R*. *sativus*, and 18 of which exhibited strong signals in comparison with those of the other chromosomes (Fig. 1H–I). This result demonstrated that the hybrid was derived from the intergeneric hybridization from *R*. *sativus* and *B*. *rapa*. In general, distant hybridization usually displays hybrid vigor, disease resistance and some other traits in the future generations^28^. However, several difficulties result from obtaining a hybrid from distant hybridization including cross incompatibility, hybrid death or sterility and progeny separation of the hybrid. Generally, except for the recombination of the nuclear genome, somatic hybridization by very distant species will lead to the hybridization of cytoplasmic organelles by protoplast fusion, which creates new genetic diversity and variations in these organelle genomes. Incorporation of whole genomes from two distant species through somatic fusion will introduce many exotic genetic materials and imbalances that induce somatic incompatibility, which might induce abnormal growth and development of the hybrid or hybrid regeneration^29^. For instance, somatic hybrids reported between the *Brassica* species in different tribes were generated by symmetric and asymmetric protoplast fusions^15^. In this study, the intergeneric hybrids with expected chromosome number and normal meiosis behavior were then sampled for further metabolomics and transcriptome analysis (Fig. 1G,J). Moreover, the intergeneric hybrid of *B*. *rapa* and *R*. *sativus* has been inherited for multiple generations and has high fertility. Therefore, the intergeneric hybrid of *B*. *rapa* and *R*. *sativus* has the potential to become a new type of vegetable and to provide valuable information to depict interactive networks between functional genes and their metabolites.

### Metabolic profiling of *B*. *rapa*, *R*. *sativus* and their hybrid

**Figure 2:**
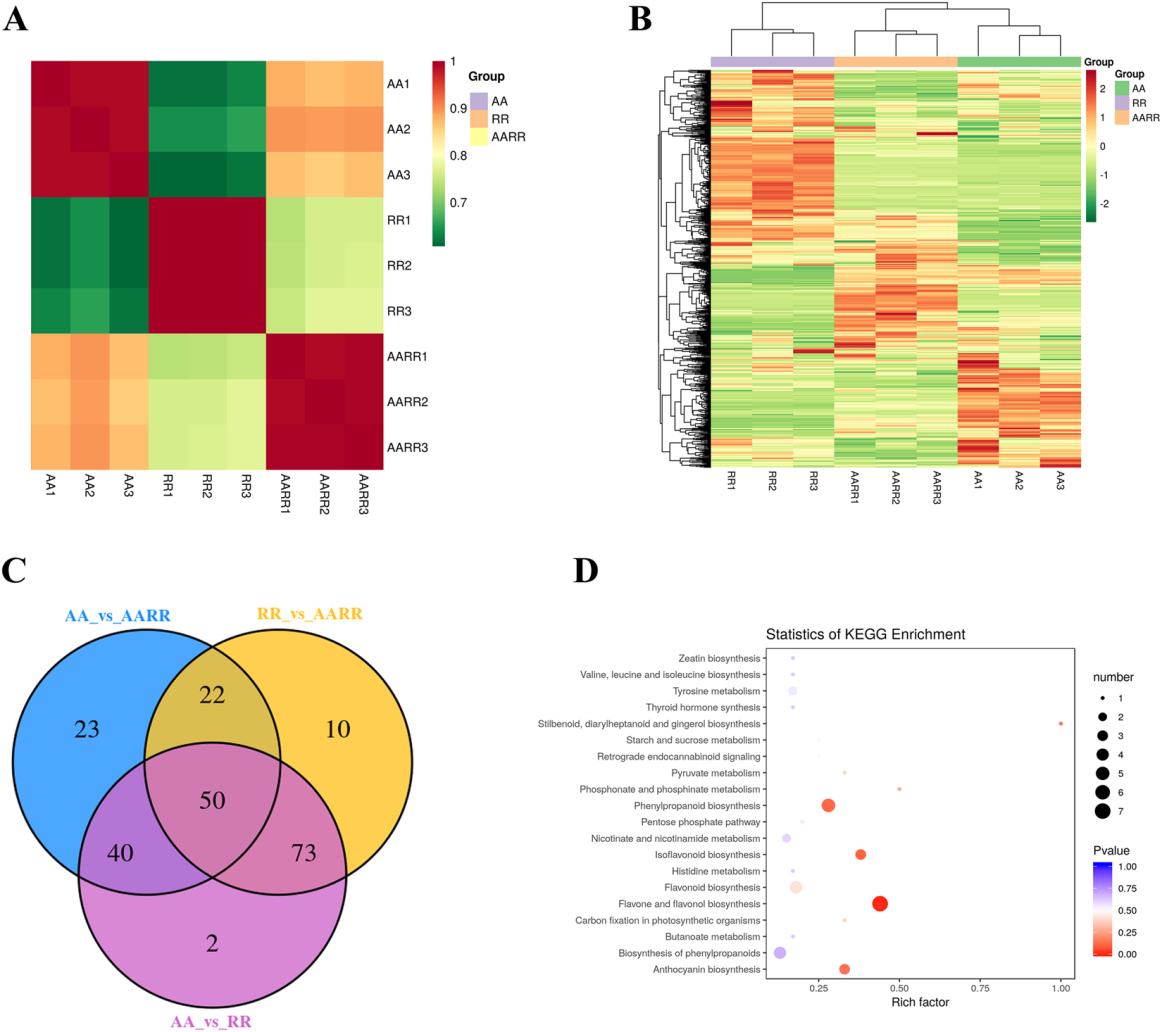
Metabolic profiling of *B*. *rapa*, *R*. *sativus* and the hybrid. (AA1, AA2 and AA3 indicate three biological replicates of AA; RR1, RR2 and RR3 indicate three biological replicates of RR; AARR1, AARR2 and AARR3 indicate three biological replicates of AARR) (**A**) The correlation coefficient analysis of sample replicates. (**B**). Heatmap analysis of overall metabolites between *B*. *rapa*, *R*. *sativus* and the hybrid. (**C**). Overlap analysis of differential metabolites between *B*. *rapa*, *R*. *sativus* and the hybrid. (**D**). KEGG analysis of differential metabolites between *B*. *rapa*, *R*. *sativus* and the hybrid.

To define a comprehensive landscape of metabolite reprogramming between *B*. *rapa*, *R*. *sativus* and their hybrid, we performed metabolite profiling of *B*. *rapa*, *R*. *sativus* and their hybrid by using LC-MS technologies. Three biological replicates of leaf tissues of *R*. *sativus*, *B*. *rapa* and the intergeneric hybrid were used for metabolic profile analysis. Figure 2A shows that the correlation coefficients of sample replicates were very high, which indicated that the metabolome data were reliable. A total of 623 metabolites were identified in *B*. *rapa*, *R*. *sativus* and their hybrid (Fig. 2B). Among the metabolites, 135 differentially expressed metabolites were detected between *B*. *rapa* and the hybrid, including 34 down-regulated and 101 up-regulated metabolites, respectively (Fig. 2C and Supplementary dataset 1). For example, many flavonoids and anthocyanins were upregulated in the hybrid. We also observed that some metabolites, including quinate derivatives and amino acid derivatives, were upregulated in the hybrid. Between *R*. *sativus* and the hybrid, 155 differentially accumulated metabolites were detected, including 76 downregulated and 79 upregulated metabolites, respectively (Fig. 2C and Supplementary dataset 1). The upregulated metabolites included flavonoids, amino acid derivatives, organic acid derivatives and so on. We also observed that 165 differentially accumulated metabolites were detected between *B*. *rapa* and *R*. *sativus*, including 68 downregulated and 97 upregulated metabolites, respectively (Fig. 2C and Supplementary dataset 1). The main differentially expressed metabolites included flavonoids, quinate derivatives, anthocyanins, coumarin derivatives, organic acid derivatives and so on. We further used the KEGG database to annotate the differential metabolites and analyze their metabolic pathways. Figure 2D lists the top 20 KEGG pathways. Among the pathways, the top 3 pathways (ranked by *p*-value) included “Flavone and flavonol biosynthesis”, “Phenylpropanoid biosynthesis” and “Isoflavonoid biosynthesis”. Further analysis showed that multiple differentially expressed flavonoids were detected between *B*. *rapa*, *R*. *sativus* and the hybrid. As shown in Supplementary dataset 2, 46 differentially expressed flavonoids were identified between *B*. *rapa* and the hybrid, including 9 downregulated and 37 upregulated flavonoids, respectively. In addition, 70 differentially expressed flavonoids were identified between *R*. *sativus* and the hybrid, including 20 downregulated and 50 upregulated flavonoids, respectively. For example, luteolin is an important flavonoid and has many potential therapeutic activities such as antioxidant, anti-inflammatory, antimicrobial and anticancer activities. The results showed that the level of luteolin of the hybrid was much higher than that of *R*. *sativus*. In addition, we found that most derivatives of luteolin were significantly accumulated in the hybrid compared with those of its parents (Supplementary dataset 2). As secondary metabolites, flavonoids are widely distributed in plants. Flavonoids are the most important plant pigments, chemical messengers, physiological regulators and cell cycle inhibitors^30^. In addition, flavonoids play an important role in plant defense^31^. Moreover, flavonoids are often used as antioxidants, as a cardioprotective, as an antihypertensive, or for blood glucose regulation^32^. *Brassica rapa* and *R*. *sativus* are two edible vegetables that contain multiple nutrients and phytochemicals including flavonoids, phenolic compounds, and anthocyanins. In this study, we explored the overall metabolite reprogramming among *B*. *rapa*, *R*. *sativus* and their hybrid and found the level of most flavonoids was significantly upregulated in the hybrid compared with that in its parents. The incorporation of whole genomes from *B*. *rapa* and *R*. *sativus* introduced numerous exotic genetic materials in the hybrid, which led to the rearrangement of the genome and induced abnormal expression of key genes involved in many metabolic pathways. Moreover, we surprisingly found that sinigrin (an important glucosinolate) was enormously upregulated in the hybrid in comparison with that in its parents (Supplementary dataset 1). Various pharmacological activities of sinigrin reveal anticancer, antibacterial, antifungal and anti-inflammatory effects^33^. For example, sinigrin inhibited bladder cancer growth and blocked muscle invasion in an orthotopic rat bladder cancer model^34^. Collectively, the abnormal expression of flavonoids and sinigrin may represent new agronomic traits in the hybrid of *B*. *rapa* and *R*. *sativus*, which indicated that the hybrid of *B*. *rapa* and *R*. *sativus* has potential to become a novel hybrid vegetable with great medicinal value in the future.

### Transcriptome profiling for *B*. *rapa*, *R*. *sativus* and their hybrid

**Figure 3:**
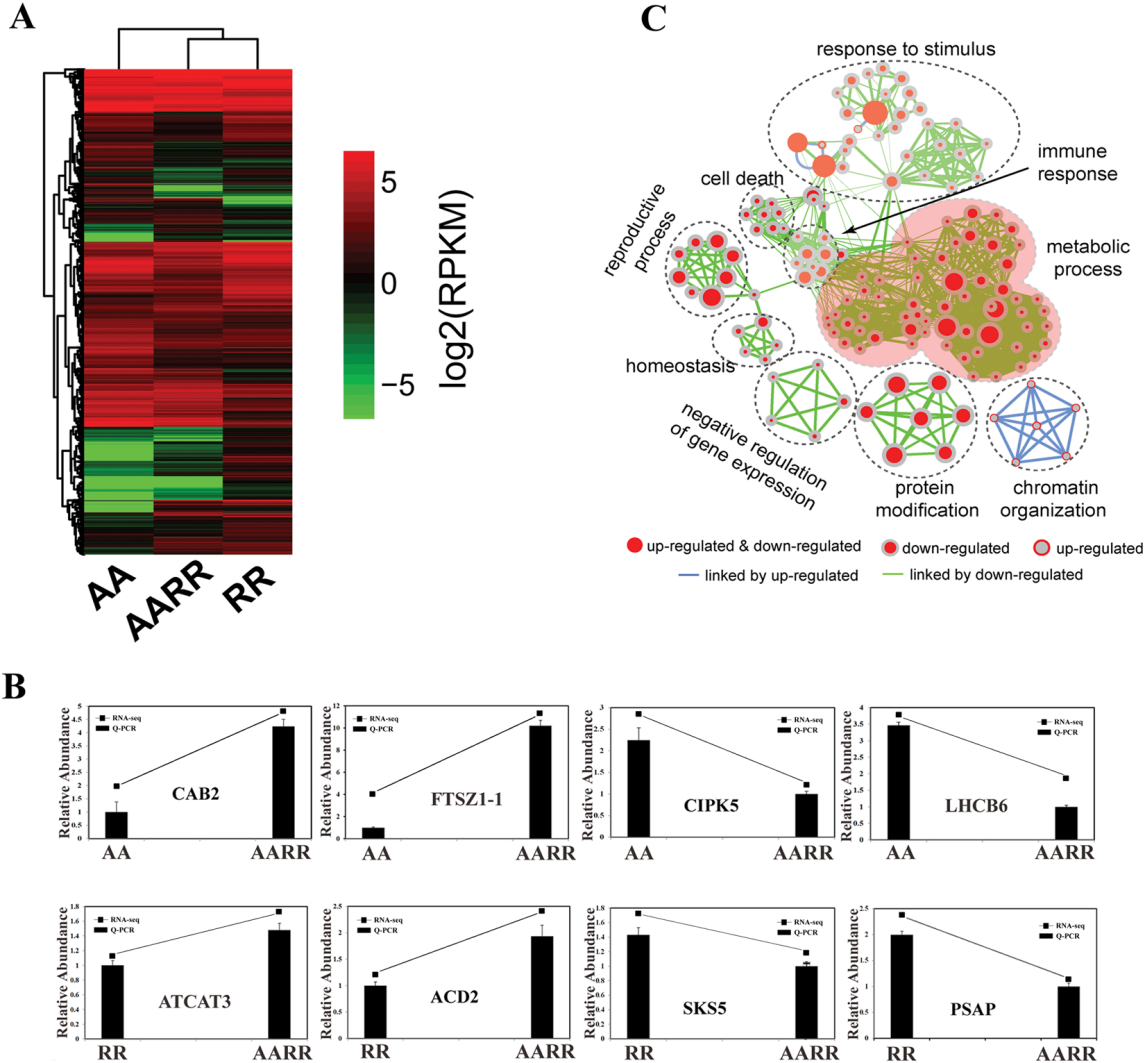
Transcriptome analysis of *B*. *rapa*, *R*. *sativus* and the hybrid. (**A**). Heatmap analysis of differentially expressed genes between *B*. *rapa*, *R*. *sativus* and the hybrid. (**B**). RNAseq analysis and quantitative RT-PCR validation of differentially expressed genes between *B*. *rapa*, *R*. *sativus* and the hybrid. (**C**). GO modules enriched with differentially expressed genes in the hybrid visualized by the EM plug-in in Cytoscape.

The leaf tissues of *R*. *sativus*, *B*. *rapa* and the intergeneric hybrid were sequenced using the Illumina Hiseq. 2000 platform. After removing the unknown reads, low-quality reads and adaptor sequences, a total of 18,873,973 and 92,858,774 and 44,155,651 clean reads were obtained for *R*. *sativus*, *B*. *rapa and the hybrid*, respectively. We conducted the *de novo* assembly of the clean reads of the hybrid from *B*. *rapa* and *R*. *sativus* by Trinity program, which generated a total of 23,671unigenes with an average length of 619 bp and an N_50_ of 668 bp (Fig S1). Furthermore, the obtained unigenes were mapped to *B*. *rapa* and *R*.*sativus* genome, respectively. The results showed that 9817 unigenes come from *B*. *rapa* and 10,631 unigenes come from *R*. *sativus*. The transcriptome differences were further examined by differential expression analysis of RNA-seq data from *B*. *rapa*, *R*. *sativus* and the hybrid using the R package edgeR. When the *B*. *rapa* genome was used as the reference, 12,643 differentially expressed genes (6155 upregulated and 6488 downregulated) were identified among *B*. *rapa*, *R*. *sativus* and the hybrid (Fig. 3A, Supplementary dataset 3). Quantitative real-time polymerase chain reaction (qRT-PCR) experiments validated the RNA-seq results using eight randomly selected differentially expressed genes (Fig. 3B).

Gene Ontology (GO) enrichment analysis was performed for the differentially expressed genes using the Cytoscape plug-in BiNGO (http://www.cytoscape.org/). Significantly enriched GO terms were clustered and visualized using the Cytoscape plug-in EnrichmentMap (EM) (http://www.cytoscape.org/). As shown in Fig. 3C, significantly enriched GO categories were clustered into nine separated modules. The largest module was the GO category of “metabolic process”, which consisted of approximately 60 daughter categories, including “small molecule metabolic process”, “glycoside metabolic process”, “purine nucleotide process”, “flavonoid metabolic process” and “metabolic process”. The other main modules contained the GO categories of “response to stimulus”, “protein modification”, “chromatin organization”, “negative regulation of gene expression”, “reproductive process”, “homeostasis”, “cell death” and “immune response”. Genome doubling reportedly influences metabolic profiles in autopolyploids. For example, Xing *et al*.^35^ found that the tetraploid *Catharanthus roseus* produced more secondary metabolites, including vindoline, catharanthine and vinblastine, than those of its diploid parents. Importantly, our gene ontology analysis showed that most differentially expressed genes were enriched in “metabolic process”. For example, we observed that several key genes involved in the flavonoid pathway, including CHI, F3H, FLS and DFR, were upregulated in the hybrid of *B*. *rapa* and *R*. *sativus* compared with those in its parents, which is consistent with the metabolome results that many flavonoids were upregulated in the hybrid in comparison with those in its parents. Taken together, Gene Ontology enrichment analysis suggested that the hybridization of *B*. *rapa* and *R*. *sativus* greatly influenced the metabolic process and activity by altering the expression level of some key genes involved in the metabolic pathways.

### Identification of differentially expressed genes involved in phenylpropanoid and flavonoid pathways

**Figure 4:**
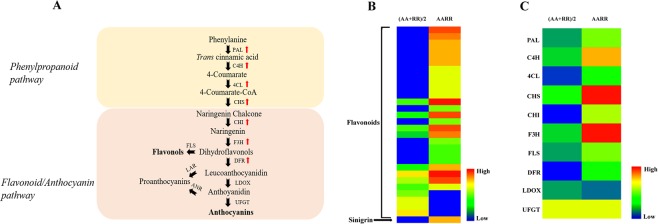
Analysis of differentially expressed genes involved in phenylpropanoid and flavonoid pathway. (**A**). The sketch map of the phenylpropanoid and flavonoid pathways. (**B**). Heatmap analysis of the differentially expressed flavonoid metabolites and sinigrin between *B*. *rapa*, *R*. *sativus* and their hybrids. (**C**). Heatmap analysis of the differentially expressed genes involved in the phenylpropanoid and flavonoid pathways.

In the process of plant growth and development, the expression level of secondary metabolites is related to special gene expression, protein modification and environmental changes. Therefore, we identified the differentially expressed genes responsible for the synthesis of secondary metabolites by the transcriptome analysis of *B*. *rapa*, *R*. *sativus* and their hybrid. The results showed that many differentially expressed flavonoids of *B*. *rapa*, *R*. *sativus* and their hybrid were detected in phenylpropanoid and flavonoid pathways (Fig. 4A,B). Therefore, we further investigated the expression level of the genes that encoded important enzymes involved in phenylpropanoid and flavonoid pathways. As shown in Fig. 4A,C, transcriptome analysis identified 10 genes involved in phenylpropanoid and flavonoid pathways. For example, some key genes in phenylpropanoid and flavonoid pathways, including PAL, C4H, 4CL, CHS, CHI, F3H, FLS and DFR, were significantly upregulated. As an upstream enzyme, C4H forms 4-coumarate to yield coenzyme A (CoA)-thioester by 4CL. 4-Coumaroyl-CoA is the precursor of flavonoid and many other phenylpropanoid compounds^36^. Previous study showed that Eucalyptus Class II C4H is involved in the pathway of stress responses and wood lignin biosynthesis, while Class I C4H is constitutively expressed in any tissue and participates in the synthesis of phenylpropanoid metabolites^37^. In addition, F3H is a 2-oxoglutarate-dependent dioxygenase and forms multi-enzyme complexes with other enzymes involved in flavonoid biosynthesis^38^. For example, the expression level of F3H is regulated by salicylic acid, jasmonic acid and abscisic acid. Moreover, the increased expression of F3H leads to the tolerance to abiotic and biotic stress including drought, cold, UV radiation, saline conditions and so on^38^. Particularly, the expression level of F3H was significantly increased over 50-fold in the hybrid compared with that in its parents. As key genes in the process of anthocyanin synthesis, the expression levels of LDOX and UFGT changed slightly among *B*. *rapa*, *R*. *sativus* and their hybrid.

Interestingly, we also observed some differentially expressed genes involved in photosynthesis in the hybrid in comparison with those of its parents. For example, the quantitative RT-PCR experiment showed that RCA was upregulated in the hybrid (Fig S2A). RCA is usually required for the light activation of the Rubisco enzyme. This result was also supported by the measurement of chlorophyll relative content by SPAD-502. As shown in Fig S2B, the average chlorophyll relative content of the hybrid, *B*. *rapa* and *R*. *sativus* was 43.6 ± 2.1, 34.4 ± 3.4 and 45.2 ± 1.5, respectively. Although the hybrid contained almost equal chlorophyll relative content to that of *R*. *sativus*, the chlorophyll content in the hybrid was significantly higher than that of *B*. *rapa*. In summary, both metabolome and transcriptome analyses showed that the intergeneric hybrid of *B*. *rapa* and *R*. *sativus* contained more health-promoting metabolites than those of its parents, suggesting the hybrid will be a valuable bioengineering resource with new agronomic traits for the improvement of vegetable varieties in the future.

## Materials and Methods

### Plant materials

*Brassica rapa* (AA, 2n = 20, Chinese cabbage, pak choi and turnip) and *R*. *sativus* (RR, 2n = 18, big root radish) were used as female parent to synthesize the intergeneric amphidiploid (AARR, 2n = 38). Chromosome doubling techniques were performed on the hybrids, and the putative amphidiploid plants with relatively normal fertility were selected for further studies. Professor Jiangsheng Wu of Huazhong Agricultural University (Professor Wu obtained AARR materials through exchange with Soo-Seong Lee of Chung-Ang University) provided the materials.

### Cytological analysis of the intergeneric hybrid

Styles of putative amphidiploid plants were used to determine the chromosome number of the intergeneric hybrids. The materials were treated with 2 mM 8-hydroxyquinoline for 4 hours at 22 °C, fixed in Carnoy for 4 hours and then stored in 70% ethanol at 4 °C before chromosome observation. The anthers were dissected out, cut in half and stained with 10%-modified carbol fuchsin for meiotic analysis^28^. DNA samples of *R*. *sativus* (RR, 2n = 18) were extracted for GISH (genomic *in situ* hybridization) analysis, which were labeled with biotin-11-dUTP (Fermentas, Lithuania) by nick translation. GISH for chromosome number and meiosis analysis were performed according to the methods described previously^39^.

### Metabolite profiling

Three biological sample sets were extracted as previously described before analysis using an LC-ESIMS/MS system^40^. The freeze-dried leaves of *B*. *rapa*, *R*. *sativus* and the hybrid were crushed using a mixer mill (MM 400, Retsch) with a zirconia bead for 1.5 min at 30 Hz. One hundred milligrams of the powder was then weighed and extracted with 1.0 ml of 70% aqueous methanol overnight at 4 °C. Following centrifugation at 10,000 g for 10 min, the extracts were absorbed and filtrated for further LC-MS analysis. The treated extracts were analyzed using an LC-ESI-MS/MS system (HPLC, Shim-pack UFLC SHIMADZU CBM30A system, www.shimadzu.com.cn/; MS, Applied Biosystems 6500 Q TRAP, www.appliedbiosystems.com.cn/). Metabolite quantification was carried out using a multiple reaction monitoring (MRM) method as previously described^41^.

### RNA-seq library construction and transcriptome sequencing

Total RNA was extracted from the same leaf tissues (in maturity stage) of *B*. *rapa*, *R*. *sativus* and the hybrid used for metabolite profiling analysis using TRIzol. mRNA was isolated from total RNA using Dynabeads oligo (dT) (Invitrogen). First- and second-strand cDNA were synthesized using Superscript II reverse transcriptase (Invitrogen) and random hexamer primers. Double-stranded cDNA was fragmented by nebulization and used to generate RNA-seq libraries as previously described^42^. The cDNA libraries were sequenced using the Illumina Hiseq. 2000 platform to produce 100 bp paired-end reads.

### qRT-PCR validation of deep sequencing

mRNA expression levels were detected in the leaves of *B*. *rapa*, *R*. *sativus* and the intergeneric hybrid using quantitative reverse transcription-PCR (qRT-PCR). For mRNA expression detection, 1 μg of total RNA was reverse-transcribed using SuperScript III reverse transcriptase (Invitrogen) and oligo (dT)18 according to the manufacturer’s protocol. The q-PCR experiment was carried out using an ABI 7300 (ABI), and each reaction was performed in triplicate. U6 RNA was set as the internal reference gene for mRNA expression detection. The primers for mRNA qRT-PCR are listed in Additional file 17: Table S1.

### Differential expression analysis

Differentially expressed mRNAs and miRNAs among *B*. *rapa* and *R*. *sativus* and the intergeneric hybrid were identified using the R package edgeR^43^ For each comparison among the three samples, differentially expressed genes were selected if the fold change was higher than two and the *p*-value was less than 0.01.

### Function enrichment analysis and visualization of genes

For a given set of genes, the statistical significance level of GO (Gene Ontology) enrichment was calculated with the Cytoscape plug-in BiNGO, based on the function annotation of homologous genes from *Arabidopsis thaliana*. A GO term was regarded significantly enriched if its Benjamini & Hochberg FDR-adjusted *p*-value was below 0.05. The enriched GO categories were visualized as a network using the Cytoscape plug-in EnrichmentMap (EM), which linked two GO categories if there were overlapping genes.

## Supplementary information

Supplementary information

Dataset 1

Dataset 2

Dataset 3

## Data Availability

The sequencing data of *B*. *rapa*, *R*. *sativus* and the hybrid supporting the results of this study were deposited in NCBI Sequence Read Archive (SRA) Sequence Database with accession number SRP065018, SRP065048 and SRP065053.
