# Author Correction: Integration of metabolome and transcriptome reveals flavonoid accumulation in the intergeneric hybrid between *Brassica rapa* and *Raphanus sativus*

**DOI:** 10.1038/s41598-020-69596-6

**Published:** 2020-07-22

**Authors:** Libin Zhang, Chuang Ma, Hongbo Chao, Yan Long, Jiangsheng Wu, Zaiyun Li, Xianhong Ge, Heng Xia, Yongtai Yin, Jacqueline Batley, Maoteng Li

**Affiliations:** 1 College of Life Science and Technology, Huazhong University of Science and Technology, Wuhan, China; 2 State Key Laboratory of Crop Stress Biology for Arid Areas, College of Life Sciences, Northwest A&F University, Yangling, China; 3 Biotechnology Research Institute, Chinese Academy of Agricultural Sciences, Beijing, China; 4 National Key Laboratory of Crop Genetic Improvement, Huazhong Agricultural University, Wuhan, China; 5 School of Plant Biology, The University of Western Australia, Crawley, Australia

Correction to: *Scientific Reports* https://doi.org/10.1038/s41598-019-54889-2, published online 04 December 2019

This Article contains errors in the Results and Discussion section under the subheading ‘Morphological and cytological analyses of the intergeneric hybrid of *B. rapa* and *R. sativus*’.

“In this study, the intergeneric hybrids with expected chromosome number and normal meiosis behavior were then sampled for further metabolomics and transcriptome analysis (Fig. 1G,J).”

should read:

“In this study, the intergeneric hybrids with expected chromosome number and normal meiosis behavior were then sampled for further metabolomics and transcriptome analysis (Fig. 1G, L)”

Additionally, the legend of Figure 1 is incorrect:

“Morphological and cytological analysis of hybrid and its parents. (**A**–**F**) The morphological comparison of leaf (**D**, in maturity stage), flower (**E**) and silique (**F**) comparison of *B*. *rapa* (Left), hybrid (Middle) and *R*. *sativus* (Right), respectively. (**G**–**I**) GISH analysis of hybrid chromosomes. 18 chromosomes were successfully hybridized with DNA of *R*. *sativus* (red signal). (**J**–**M**) The meiosis analysis of hybrid ((**G,H,J**) were the cells with abnormal meiosis behavior, I was the cell with normal meiosis behavior).”

should read:

“Morphological and cytological analysis of hybrid and its parents. (**A**–**F**). The morphological comparison of leaf (**D**, in maturity stage), flower (**E**) and silique (**F**) comparison of *B*. *rapa* (Left), hybrid (Middle) and *R*. *sativus* (Right), respectively. (**G**–**I**). GISH analysis of hybrid chromosomes. 18 chromosomes were successfully hybridized with DNA of *R*. *sativus* (red signal). (**J**–**M**). The meiosis analysis of hybrid (**J,K,M**) were the cells with abnormal meiosis behavior, **L** was the cell with normal meiosis behavior).

