# Supplementary material for: Integration of metabolome and transcriptome reveals flavonoid accumulation in the intergeneric hybrid between Brassica rapa and Raphanus sativus
Source: Sci Rep. 2019 Dec 4;9:18368. doi: 10.1038/s41598-019-54889-2 (PMC6893016; doi:10.1038/s41598-019-54889-2)
Supplement: Supplementary file 1 — Supplementary information [file 41598_2019_54889_MOESM1_ESM.pdf]

Integration of metabolome and transcriptome reveals flavonoid accumulation in the intergeneric hybrid between *Brassica rapa* and *Raphanus sativus*

Libin Zhang[1†], Chuang Ma[2†], Hongbo Chao[1], Yan Long[3], Jiangsheng Wu[4], Zaiyun Li[4], Xianhong Ge[4], Heng Xia[1], Yongtai Yin[1], Jacqueline Batley[5] and Maoteng Li[1]*

[1]College of Life Science and Technology, Huazhong University of Science and Technology, Wuhan, China

[2]State Key Laboratory of Crop Stress Biology for Arid Areas, College of Life Sciences, Northwest A&F University, Yangling, China

[3]Biotechnology Research Institute, Chinese Academy of Agricultural Sciences, Beijing, China

[4]National Key Laboratory of Crop Genetic Improvement, Huazhong Agricultural University, Wuhan, China

[5]School of Plant Biology, The University of Western Australia, Crawley, Australia

†These authors have contributed equally to this work

* Corresponding Author: E-mail: limaoteng426@hust.edu.cn

Supplementary Information: This file contains 2 supplementary figures and 1 supplementary table.

Figure legends:

Fig S1: The *de novo* assembly result of the hybrid transcriptome.

Fig S2. (A). qRT-PCR validation of RCA mRNA. (B). The chlorophyll content analysis of *B. rapa*, *R. sativus* and the hybrid.

Fig S1.

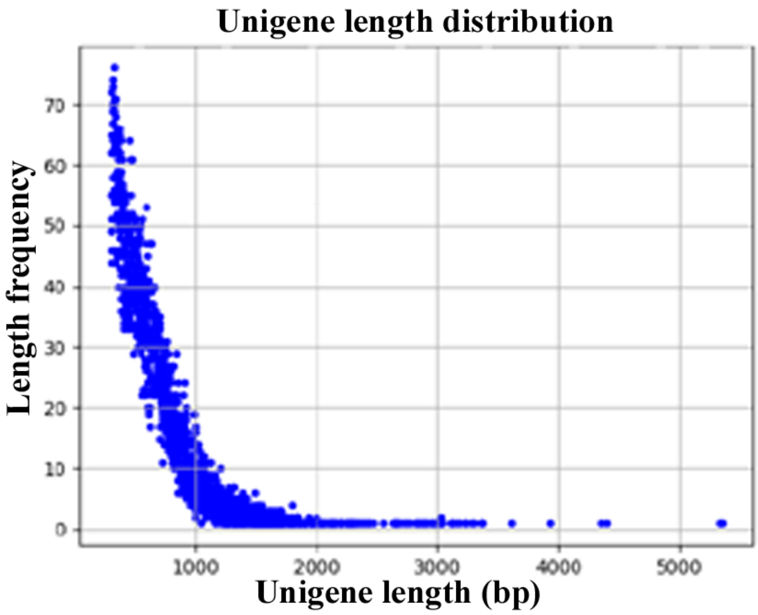

Fig S2.

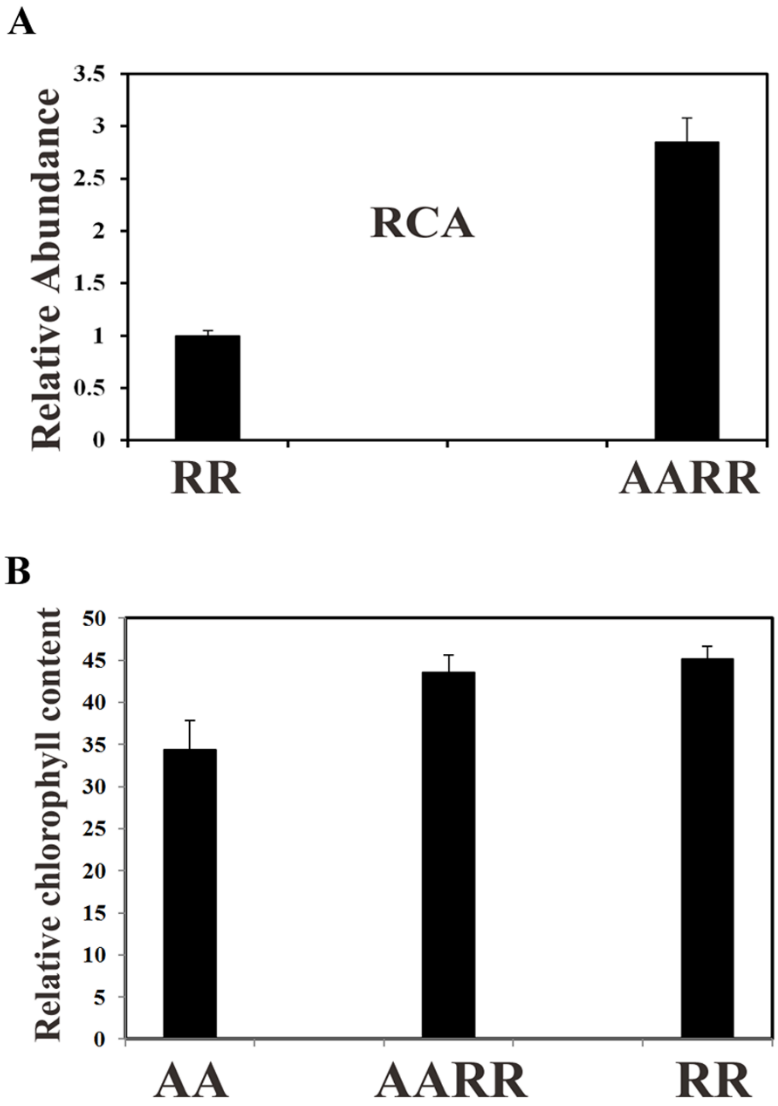
A
3.5
3
2.5
2
1.5
1
0.5
0
Relative Abundance
RCA
RR
AARR
B
50
45
40
35
30
25
20
15
10
5
0
Relative chlorophyll content
AA
AARR
RR

Table S1.

Table S1: Primers for qRT-PCR validation of differentially expressed mRNAs.		
**mRNA**	**Forward primer (5’→3’)**	**Reverse primer (5’→3’)**
ACD2	TACAGGGAAGGAGTTGCGAG	TGTCCGAGCTCAATTCCAGT
ATCAT3	TGGTGTAAAACTTGACGGCG	TTCACGCCAGAGGAATCAGT
FTSZ1-1	TCGGGGTAGGTGTTTCTTCC	GAATCTCTCCGGTGTAGCGA
CIPK5	TCGCGATCTGAAACCTGAGA	TCTTTAATACCTCCGGCGCA
PSAP	TCCGGTAACTCTGTACTCGC	TTCCCTTCCGACACTTCCTC
SKS5	GCTCTAAAACGGTCTGGTGC	GATCCGAGTTTTGGGCAAGG
LHCB6	GGTCGATTTCTTCAACCCGG	TTCAGCCTCTCCAGCTTCTC
CAB2	CTGGAGACTACGGATGGGAC	AGTAATCGAGCCCTCCATCG
U6	CGGGGACATCCGATAAAATTGG	TCTCGATTTATGCGTGTCATCCT
